# Supplementary material for: Past rapid warmings as a constraint on greenhouse-gas climate feedbacks
Source: Commun Earth Environ. 2022 Aug 30;3(1):196. doi: 10.1038/s43247-022-00536-0 (PMC11757147; doi:10.1038/s43247-022-00536-0)
Supplement: Supplementary file 2 — Supplementary Information [file 43247_2022_536_MOESM2_ESM.pdf]

Supplementary Information

Past rapid warmings as a constraint on greenhouse-gas climate feedbacks

Target journal: Communications Earth & Environment

Type: Article

Authors:

Mengmeng Liu[1,*], Iain Colin Prentice[1,2,3], Laurie Menviel[4], Sandy P. Harrison[3,5]

1: Department of Life Sciences, Imperial College London, Silwood Park Campus, Buckhurst Road, Ascot SL5 7PY, UK

2: Department of Biological Sciences, Macquarie University, North Ryde, NSW 2109, Australia

3: Ministry of Education Key Laboratory for Earth System Modelling, Department of Earth System Science, Tsinghua University, Beijing 100084, China

4: Climate Change Research Centre/ESSRC, The University of New South Wales, Sydney, Australia

5: Department of Geography and Environmental Science, University of Reading, Reading, RG6 6AB, UK

*** Corresponding author: m.liu18@imperial.ac.uk**

Supplementary Figures

1. Identify minimum and maximum

Supplementary Fig. 1.1. Minimum and maximum of (a) CO_2 concentration, (b) CH_4 concentration, (c) N_2O concentration and (d) global mean temperature anomaly to 30 ka, identified for D-O event 5. The age is on AICC2012 timescale (BP 1950) for CO_2, CH_4 and N_2O, and on absolute timescale for global mean temperature anomaly to 30 ka. The vertical black lines show the official start date of D-O warming events (on AICC2012 timescale), with numbers indicating which events they are. When there is a shift of start date, the actual start date is indicated by a red vertical dashed line. The horizontal dashed lines show the minimum and maximum identified. The grey shades show the corresponding ages from the minimum to the maximum, with text in the middle showing the length of the shades. The red line segments indicate the standard errors of the minimum and maximum. The measurements of CH_4 concentration are very accurate so the vertical line segments are small and not observable on these plots. All the data are binned in 25 years.

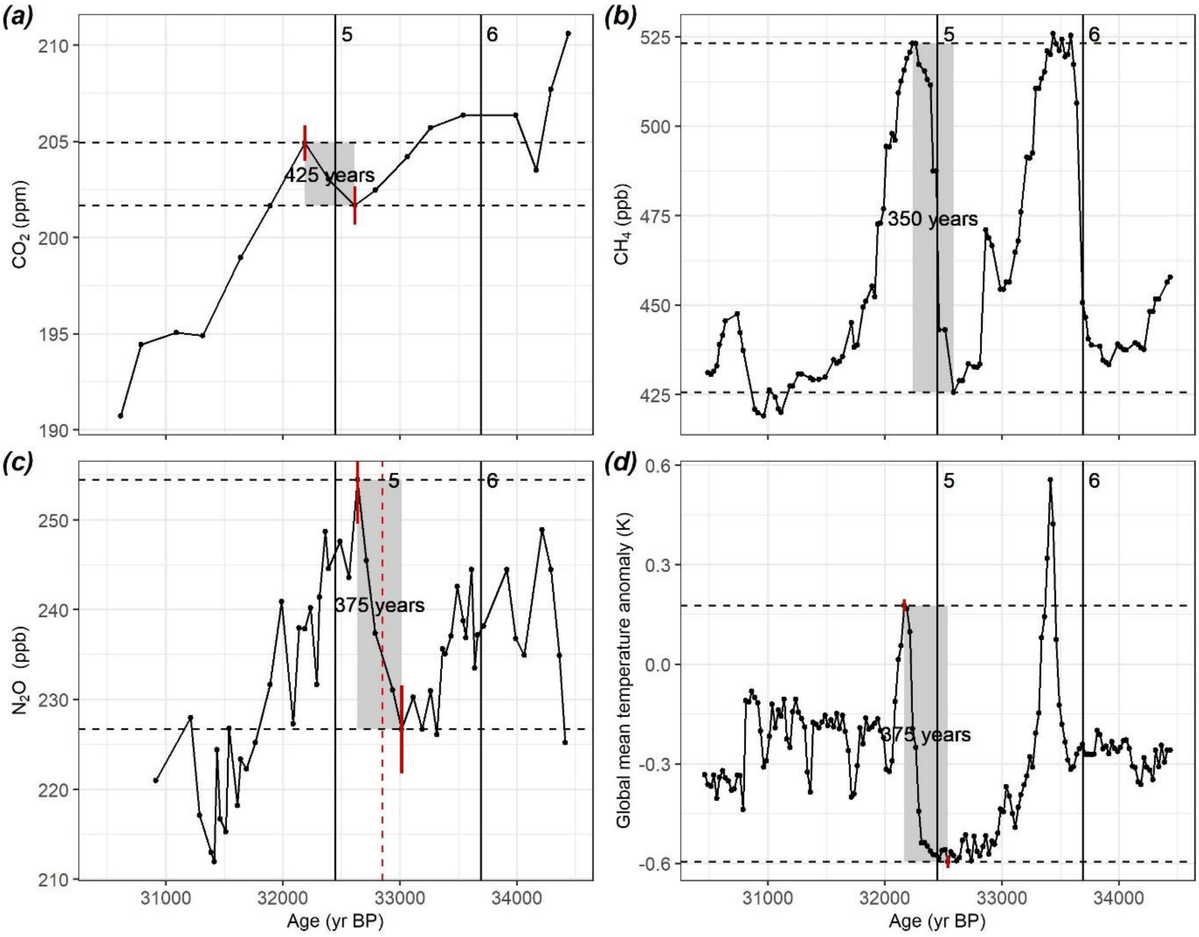

Supplementary Fig. 1.2. Minimum and maximum of (a) CO_2 concentration, (b) CH_4 concentration, (c) N_2O concentration and (d) global mean temperature anomaly to 30 ka, identified for D-O event 6. The age is on AICC2012 timescale (BP 1950) for CO_2, CH_4 and N_2O, and on absolute timescale for global mean temperature anomaly to 30 ka. The vertical black lines show the official start date of D-O warming events (on AICC2012 timescale), with numbers indicating which events they are. When there is a shift of start date, the actual start date is indicated by a red vertical dashed line. The horizontal dashed lines show the minimum and maximum identified. The grey shades show the corresponding ages from the minimum to the maximum, with text in the middle showing the length of the shades. The red line segments indicate the standard errors of the minimum and maximum. The measurements of CH_4 concentration are very accurate so the vertical line segments are small and not observable on these plots. All the data are binned in 25 years.

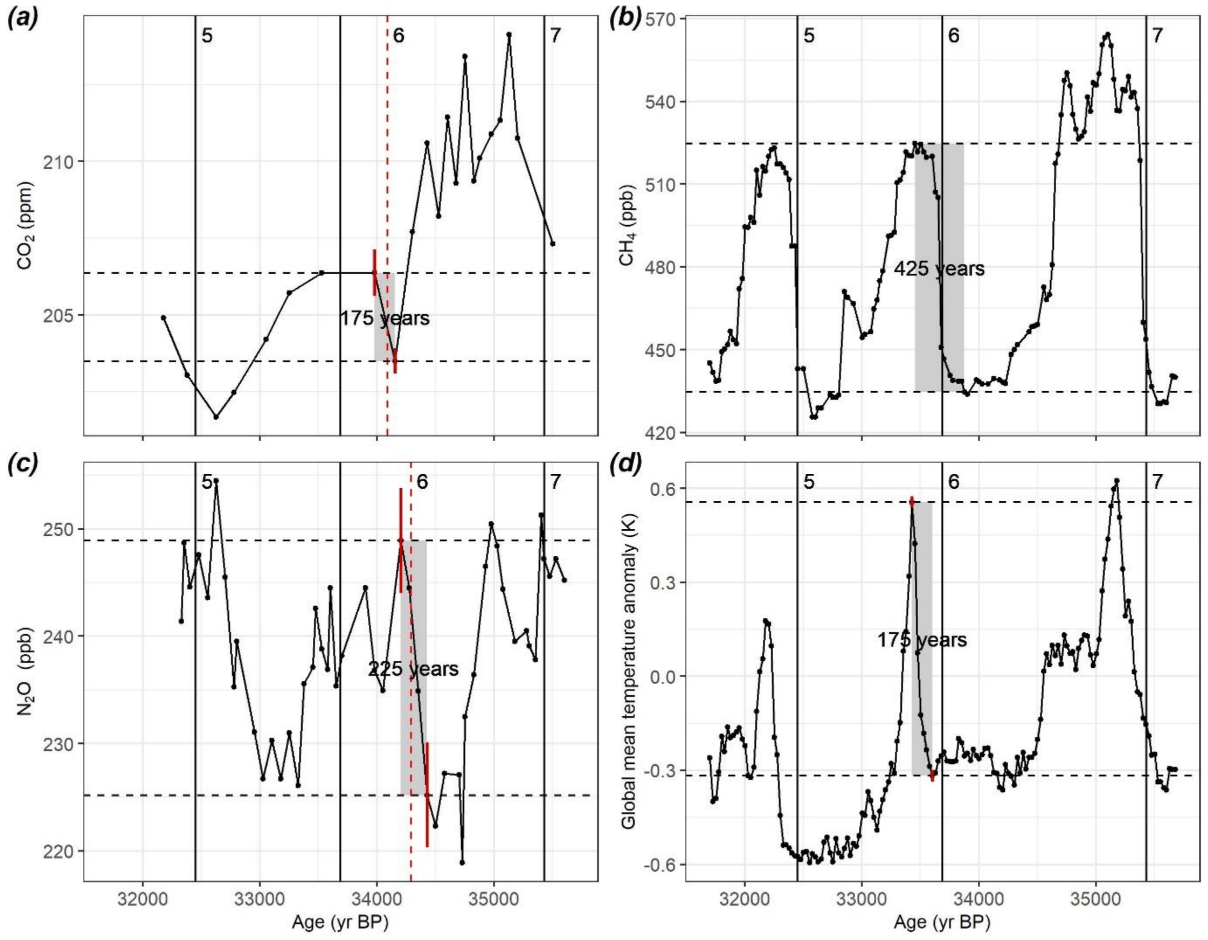

Supplementary Fig. 1.3. Minimum and maximum of (a) CO_2 concentration, (b) CH_4 concentration, (c) N_2O concentration and (d) global mean temperature anomaly to 30 ka, identified for D-O event 7. The age is on AICC2012 timescale (BP 1950) for CO_2, CH_4 and N_2O, and on absolute timescale for global mean temperature anomaly to 30 ka. The vertical black lines show the official start date of D-O warming events (on AICC2012 timescale), with numbers indicating which events they are. When there is a shift of start date, the actual start date is indicated by a red vertical dashed line. The horizontal dashed lines show the minimum and maximum identified. The grey shades show the corresponding ages from the minimum to the maximum, with text in the middle showing the length of the shades. The red line segments indicate the standard errors of the minimum and maximum. The measurements of CH_4 concentration are very accurate so the vertical line segments are small and not observable on these plots. All the data are binned in 25 years.

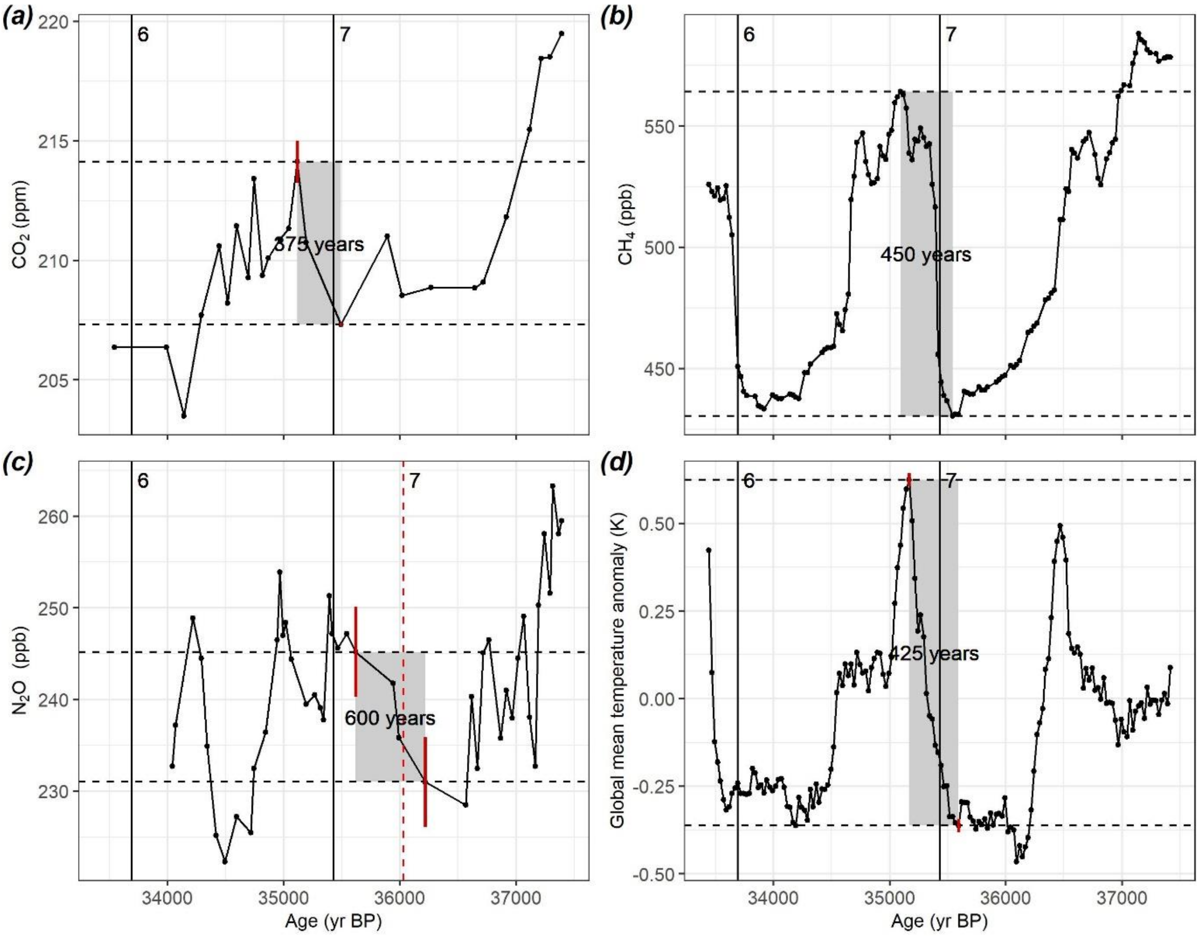

Supplementary Fig. 1.4. Minimum and maximum of (a) CO_2 concentration, (b) CH_4 concentration, (c) N_2O concentration and (d) global mean temperature anomaly to 30 ka, identified for D-O event 8. The age is on AICC2012 timescale (BP 1950) for CO_2, CH_4 and N_2O, and on absolute timescale for global mean temperature anomaly to 30 ka. The vertical black lines show the official start date of D-O warming events (on AICC2012 timescale), with numbers indicating which events they are. When there is a shift of start date, the actual start date is indicated by a red vertical dashed line. The horizontal dashed lines show the minimum and maximum identified. The grey shades show the corresponding ages from the minimum to the maximum, with text in the middle showing the length of the shades. The red line segments indicate the standard errors of the minimum and maximum. The measurements of CH_4 concentration are very accurate so the vertical line segments are small and not observable on these plots. All the data are binned in 25 years.

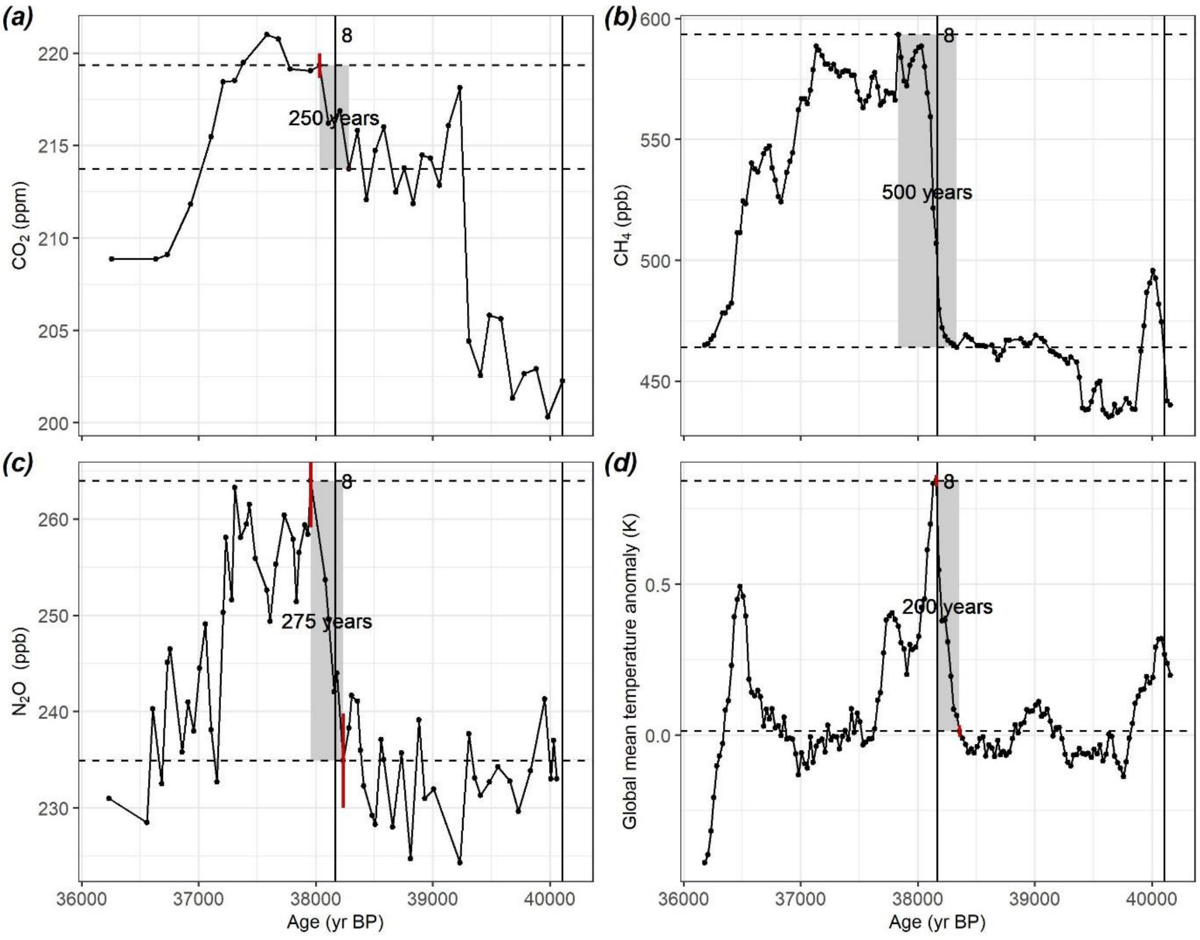

Supplementary Fig. 1.5. Minimum and maximum of (a) CO_2 concentration, (b) CH_4 concentration, (c) N_2O concentration and (d) global mean temperature anomaly to 30 ka, identified for D-O event 9. The age is on AICC2012 timescale (BP 1950) for CO_2, CH_4 and N_2O, and on absolute timescale for global mean temperature anomaly to 30 ka. The vertical black lines show the official start date of D-O warming events (on AICC2012 timescale), with numbers indicating which events they are. When there is a shift of start date, the actual start date is indicated by a red vertical dashed line. The horizontal dashed lines show the minimum and maximum identified. The grey shades show the corresponding ages from the minimum to the maximum, with text in the middle showing the length of the shades. The red line segments indicate the standard errors of the minimum and maximum. The measurements of CH_4 concentration are very accurate so the vertical line segments are small and not observable on these plots. All the data are binned in 25 years.

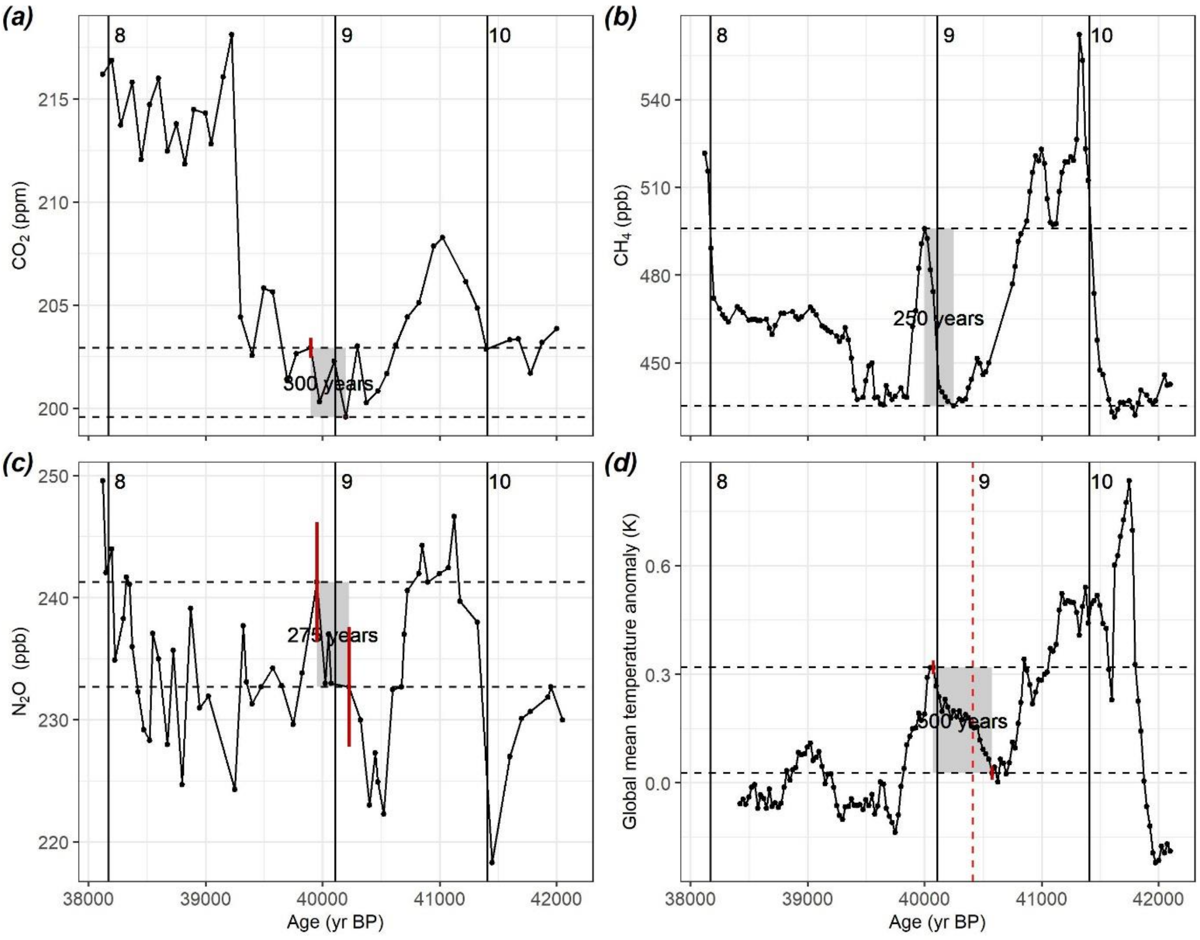

Supplementary Fig. 1.6. Minimum and maximum of (a) CO_2 concentration, (b) CH_4 concentration, (c) N_2O concentration and (d) global mean temperature anomaly to 30 ka, identified for D-O event 10. The age is on AICC2012 timescale (BP 1950) for CO_2, CH_4 and N_2O, and on absolute timescale for global mean temperature anomaly to 30 ka. The vertical black lines show the official start date of D-O warming events (on AICC2012 timescale), with numbers indicating which events they are. When there is a shift of start date, the actual start date is indicated by a red vertical dashed line. The horizontal dashed lines show the minimum and maximum identified. The grey shades show the corresponding ages from the minimum to the maximum, with text in the middle showing the length of the shades. The red line segments indicate the standard errors of the minimum and maximum. The measurements of CH_4 concentration are very accurate so the vertical line segments are small and not observable on these plots. All the data are binned in 25 years.

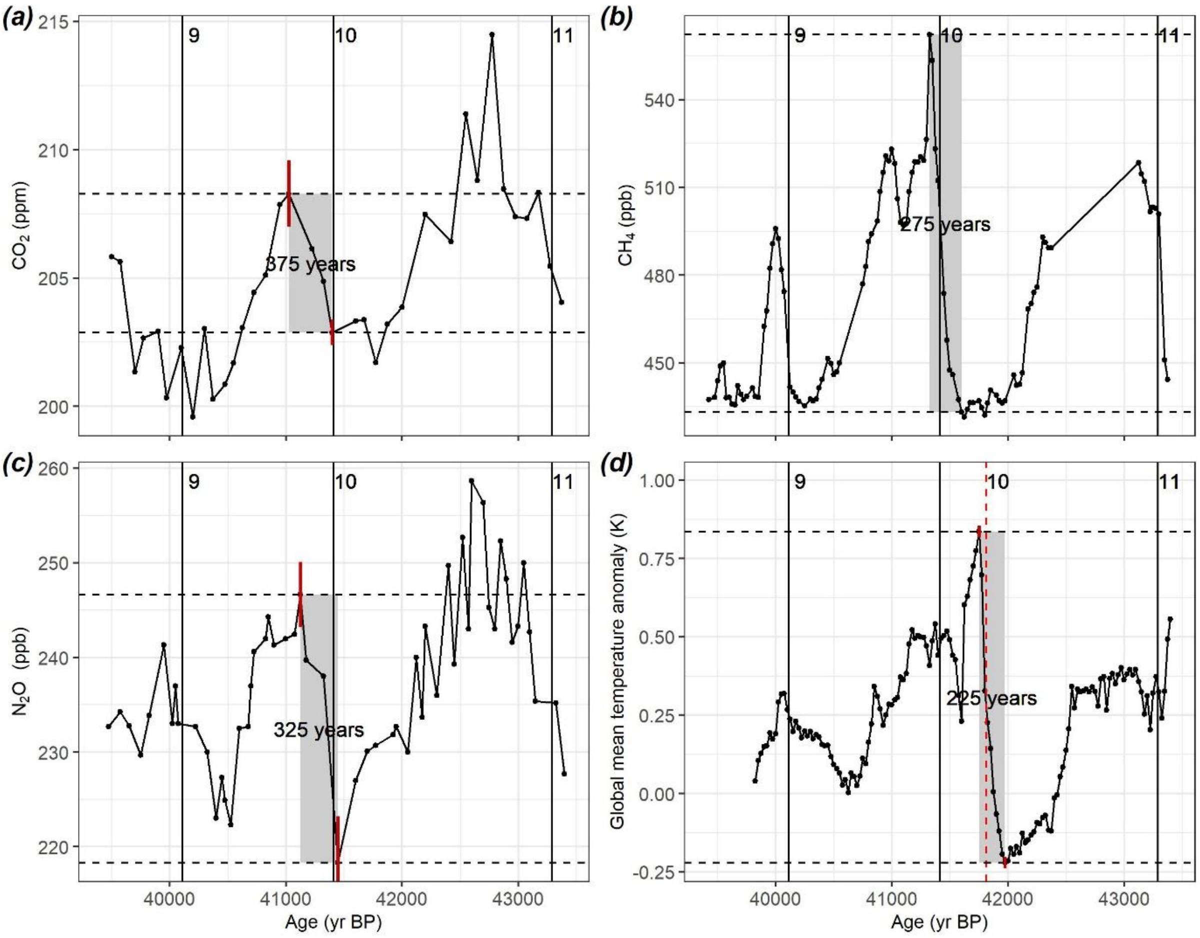

Supplementary Fig. 1.7. Minimum and maximum of (a) CO_2 concentration, (b) CH_4 concentration, (c) N_2O concentration and (d) global mean temperature anomaly to 30 ka, identified for D-O event 11. The age is on AICC2012 timescale (BP 1950) for CO_2, CH_4 and N_2O, and on absolute timescale for global mean temperature anomaly to 30 ka. The vertical black lines show the official start date of D-O warming events (on AICC2012 timescale), with numbers indicating which events they are. When there is a shift of start date, the actual start date is indicated by a red vertical dashed line. The horizontal dashed lines show the minimum and maximum identified. The grey shades show the corresponding ages from the minimum to the maximum, with text in the middle showing the length of the shades. The red line segments indicate the standard errors of the minimum and maximum. The measurements of CH_4 concentration are very accurate so the vertical line segments are small and not observable on these plots. All the data are binned in 25 years.

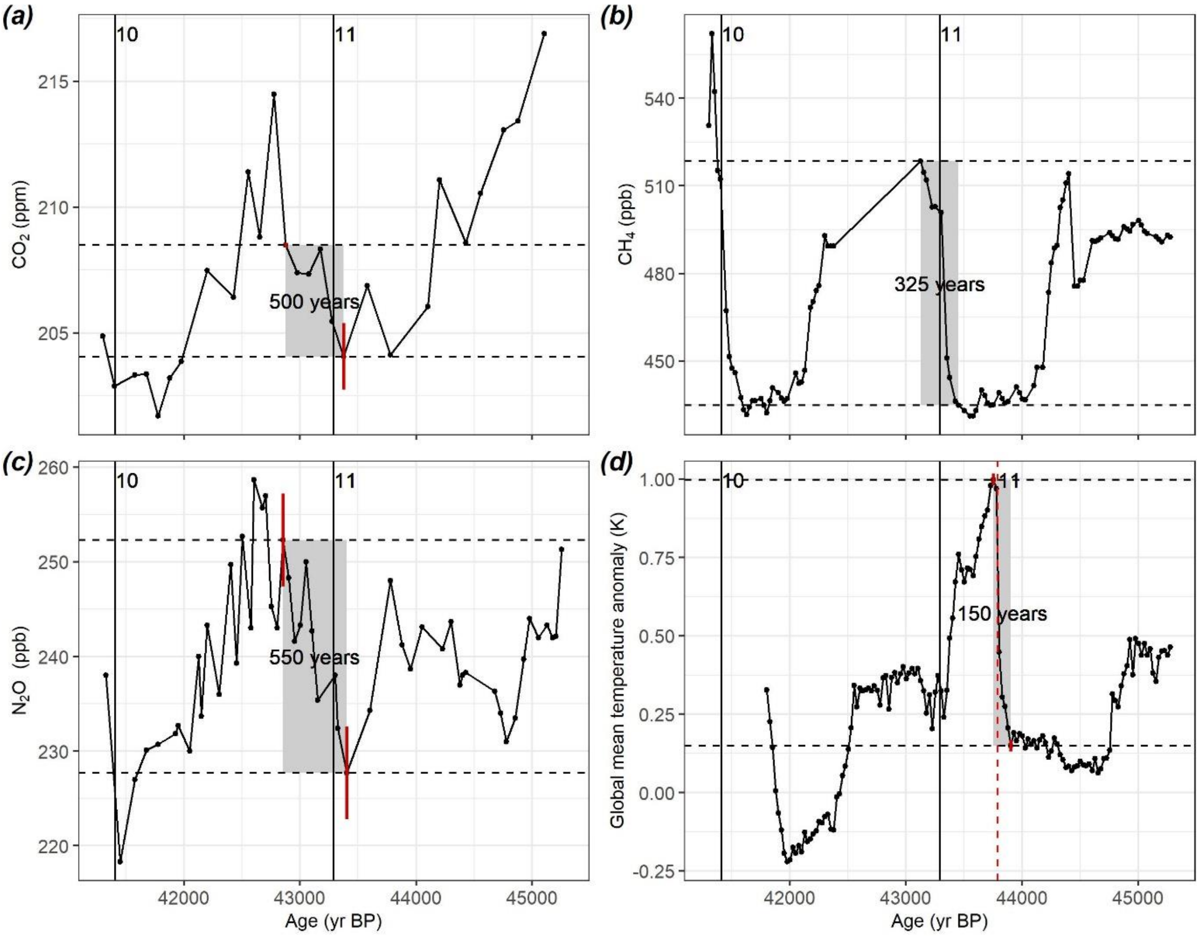

Supplementary Fig. 1.8. Minimum and maximum of (a) CO_2 concentration, (b) CH_4 concentration, (c) N_2O concentration and (d) global mean temperature anomaly to 30 ka, identified for D-O event 12. The age is on AICC2012 timescale (BP 1950) for CO_2, CH_4 and N_2O, and on absolute timescale for global mean temperature anomaly to 30 ka. The vertical black lines show the official start date of D-O warming events (on AICC2012 timescale), with numbers indicating which events they are. When there is a shift of start date, the actual start date is indicated by a red vertical dashed line. The horizontal dashed lines show the minimum and maximum identified. The grey shades show the corresponding ages from the minimum to the maximum, with text in the middle showing the length of the shades. The red line segments indicate the standard errors of the minimum and maximum. The measurements of CH_4 concentration are very accurate so the vertical line segments are small and not observable on these plots. All the data are binned in 25 years.

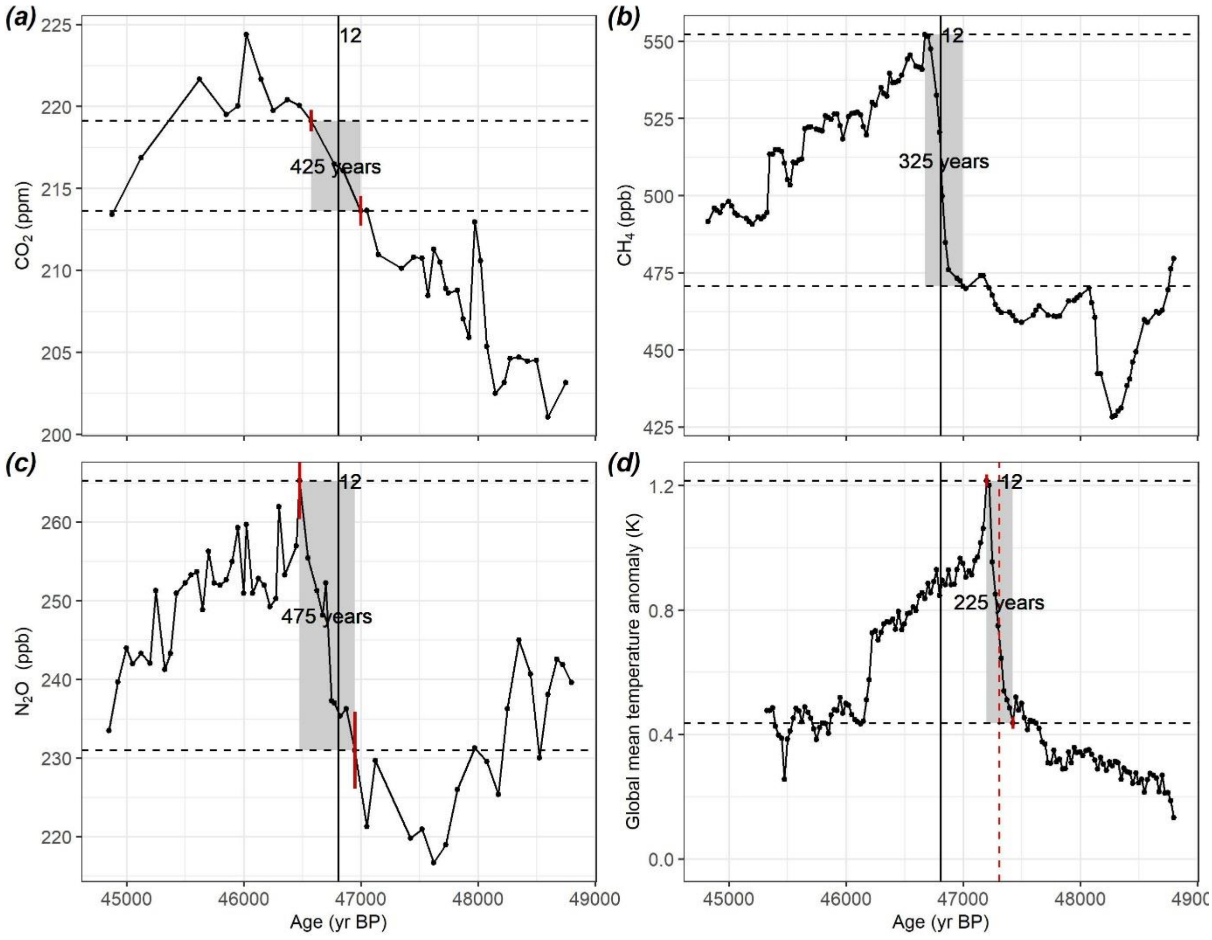

2. Get global mean temperature change

Supplementary Fig. 2.1. Comparison of simulated and observed temperature change during D-O event 5. The coloured map shows the simulated global temperature change pattern. The arrow shows the observed trend (warming or cooling) during all D-O cycles in the record. The text (if any) shows the quantitative temperature change for each D-O cycle in the record.

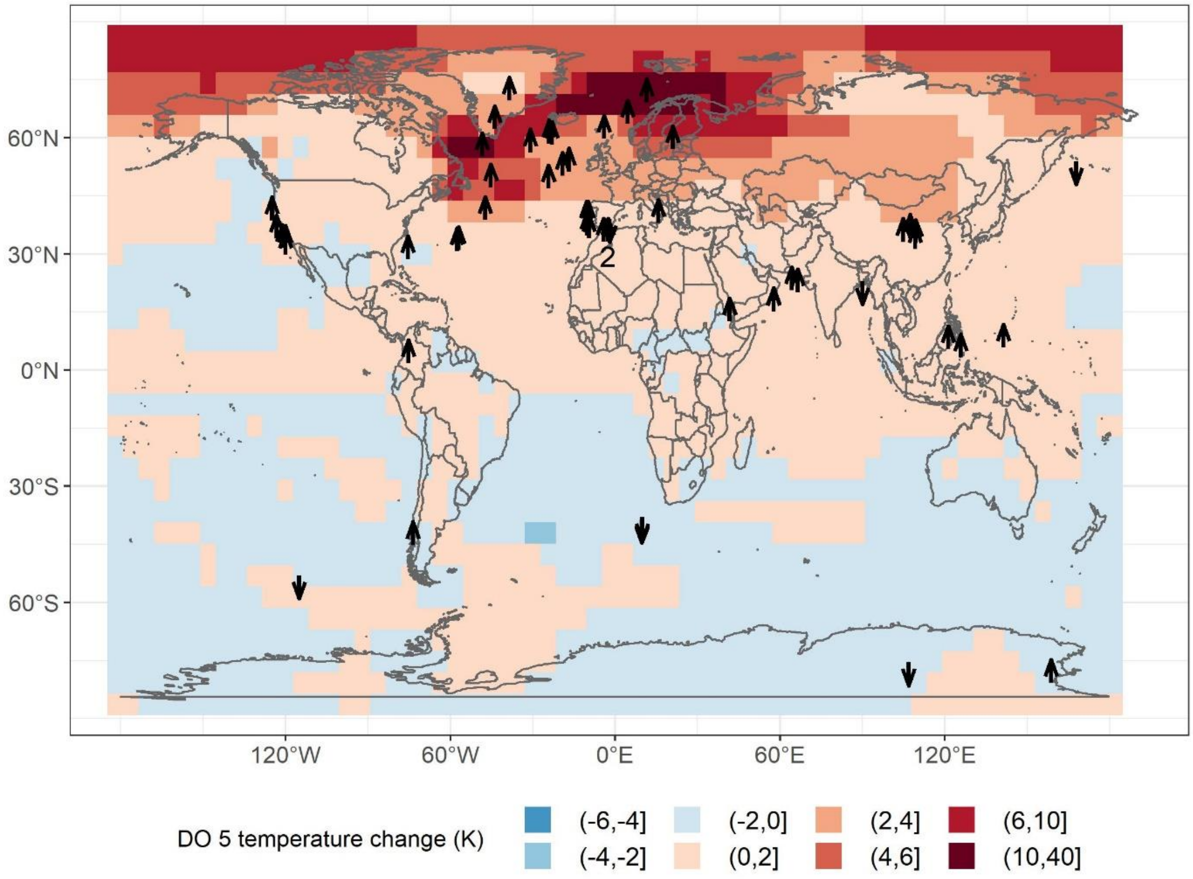

Supplementary Fig. 2.2. Comparison of simulated and observed temperature change during D-O event 6. The coloured map shows the simulated global temperature change pattern. The arrow shows the observed trend (warming or cooling) during all D-O cycles in the record. The text (if any) shows the quantitative temperature change for each D-O cycle in the record.

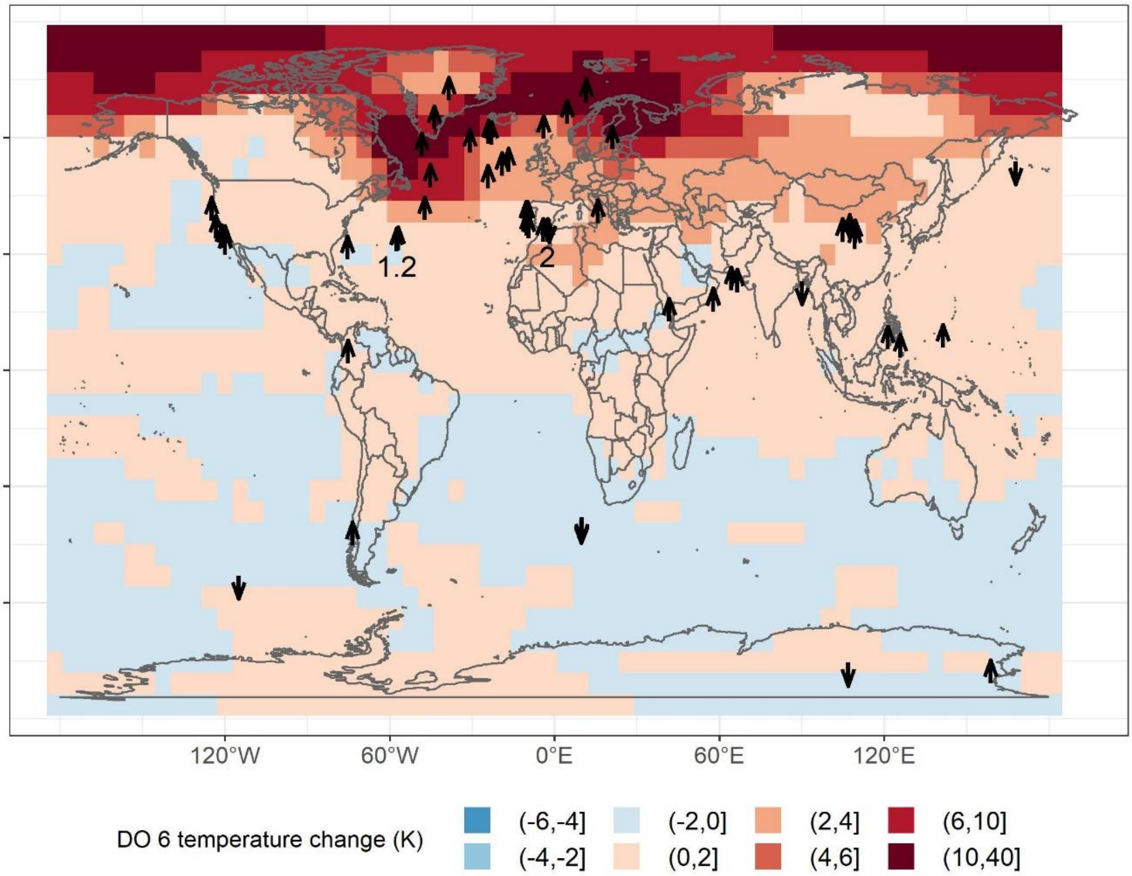

Supplementary Fig. 2.3. Comparison of simulated and observed temperature change during D-O event 7. The coloured map shows the simulated global temperature change pattern. The arrow shows the observed trend (warming or cooling) during all D-O cycles in the record. The text (if any) shows the quantitative temperature change for each D-O cycle in the record.

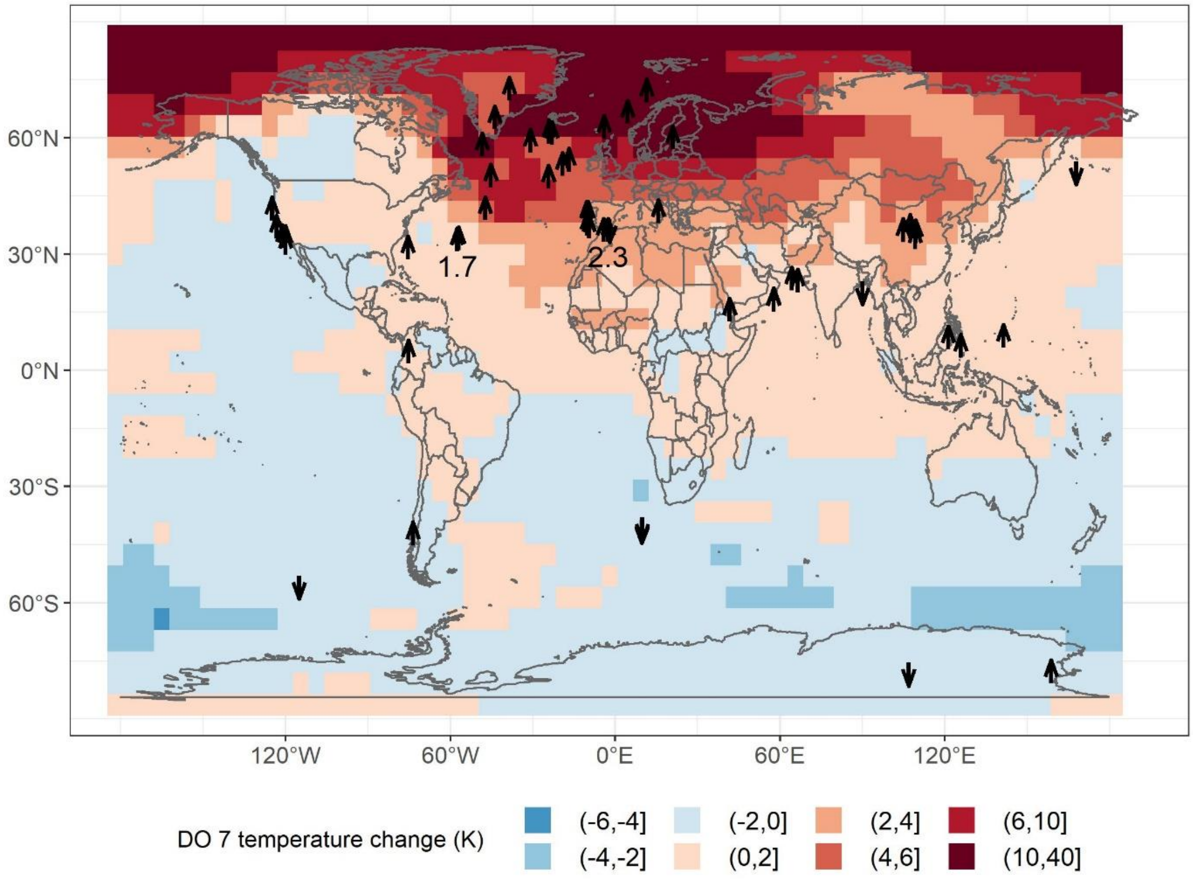

Supplementary Fig. 2.4. Comparison of simulated and observed temperature change during D-O event 8. The coloured map shows the simulated global temperature change pattern. The arrow shows the observed trend (warming or cooling) during all D-O cycles in the record. The text (if any) shows the quantitative temperature change for each D-O cycle in the record.

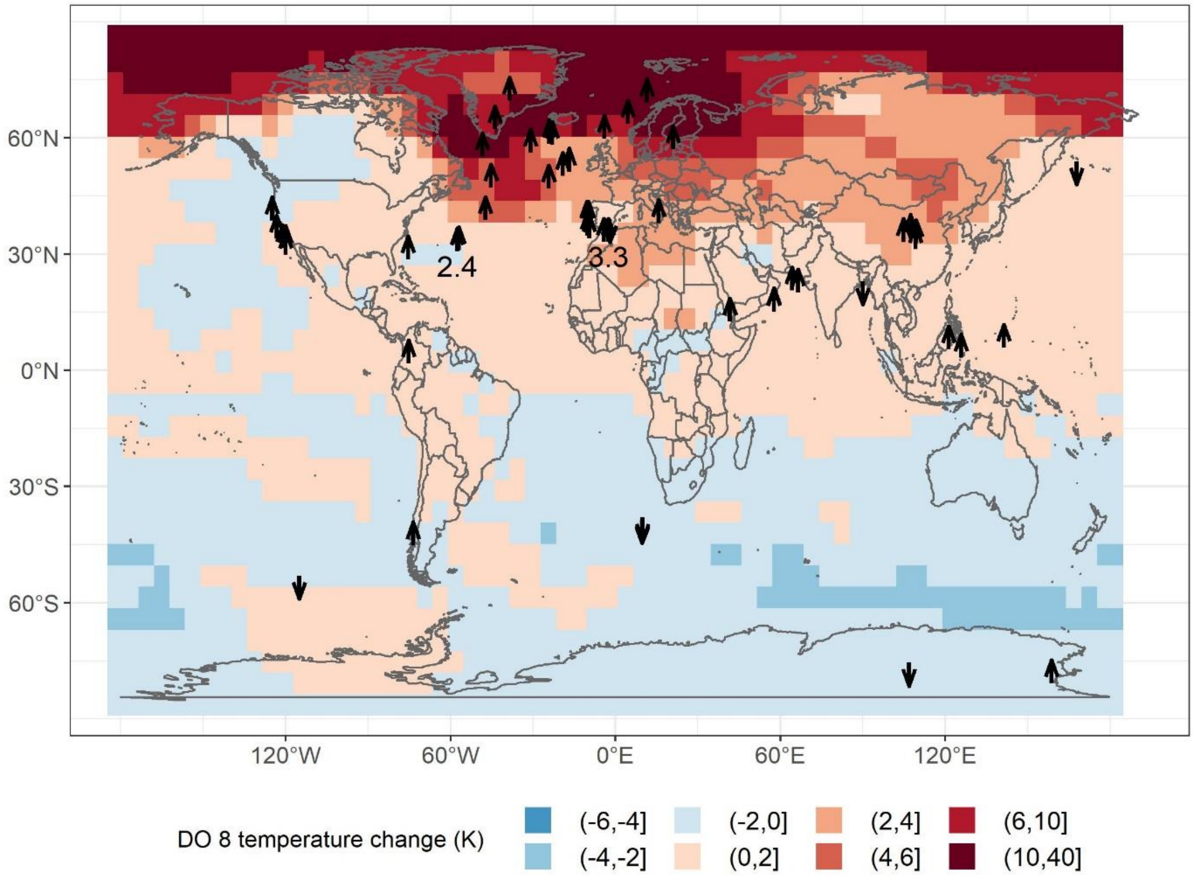

Supplementary Fig. 2.5. Comparison of simulated and observed temperature change during D-O event 9. The coloured map shows the simulated global temperature change pattern. The arrow shows the observed trend (warming or cooling) during all D-O cycles in the record. The text (if any) shows the quantitative temperature change for each D-O cycle in the record.

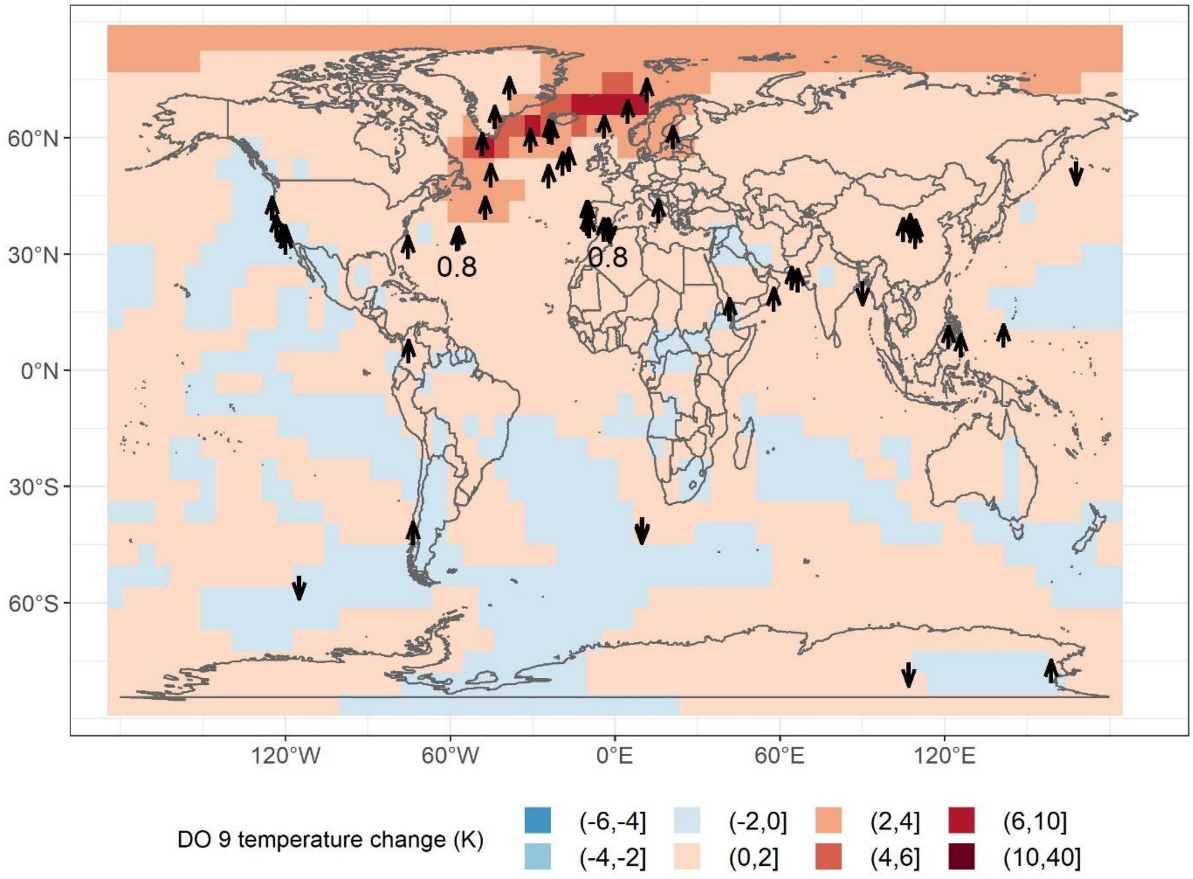

Supplementary Fig. 2.6. Comparison of simulated and observed temperature change during D-O event 10. The coloured map shows the simulated global temperature change pattern. The arrow shows the observed trend (warming or cooling) during all D-O cycles in the record. The text (if any) shows the quantitative temperature change for each D-O cycle in the record.

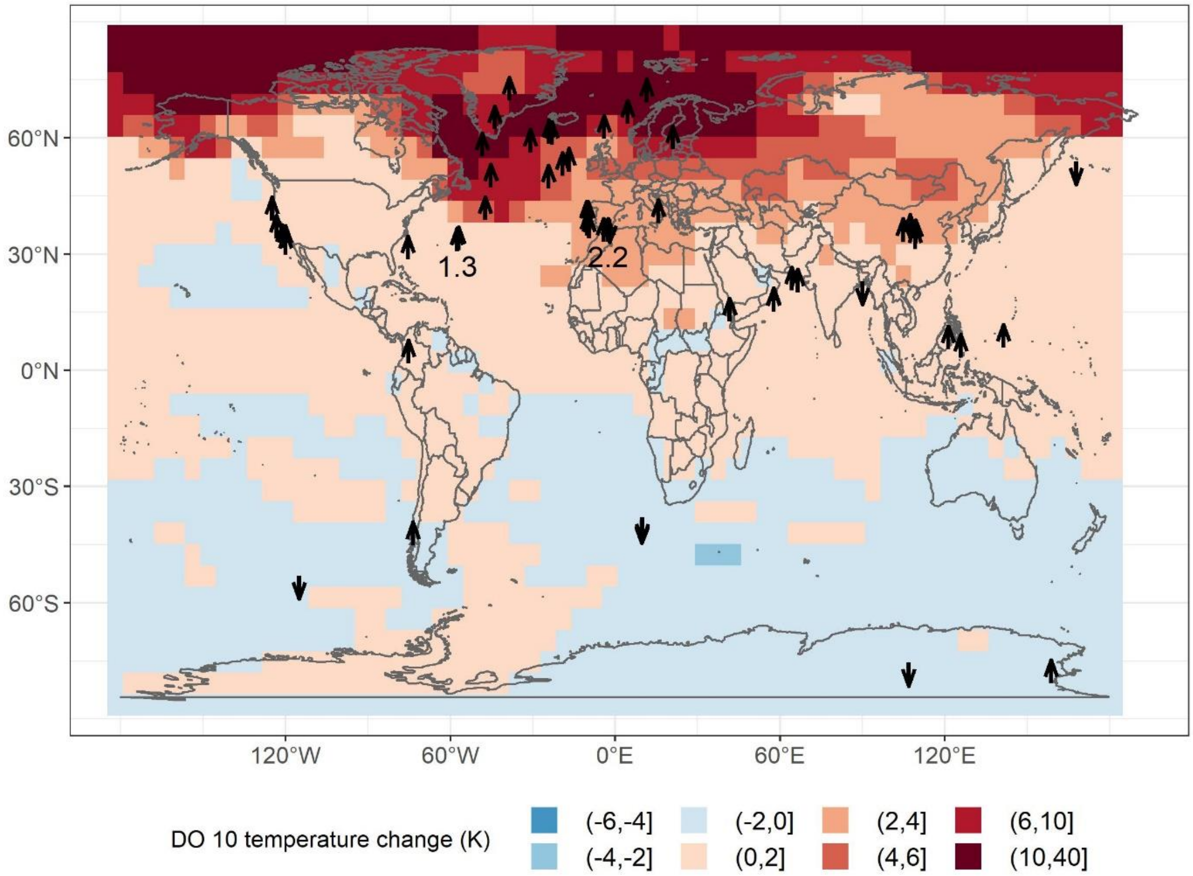

Supplementary Fig. 2.7. Comparison of simulated and observed temperature change during D-O event 11. The coloured map shows the simulated global temperature change pattern. The arrow shows the observed trend (warming or cooling) during all D-O cycles in the record. The text (if any) shows the quantitative temperature change for each D-O cycle in the record.

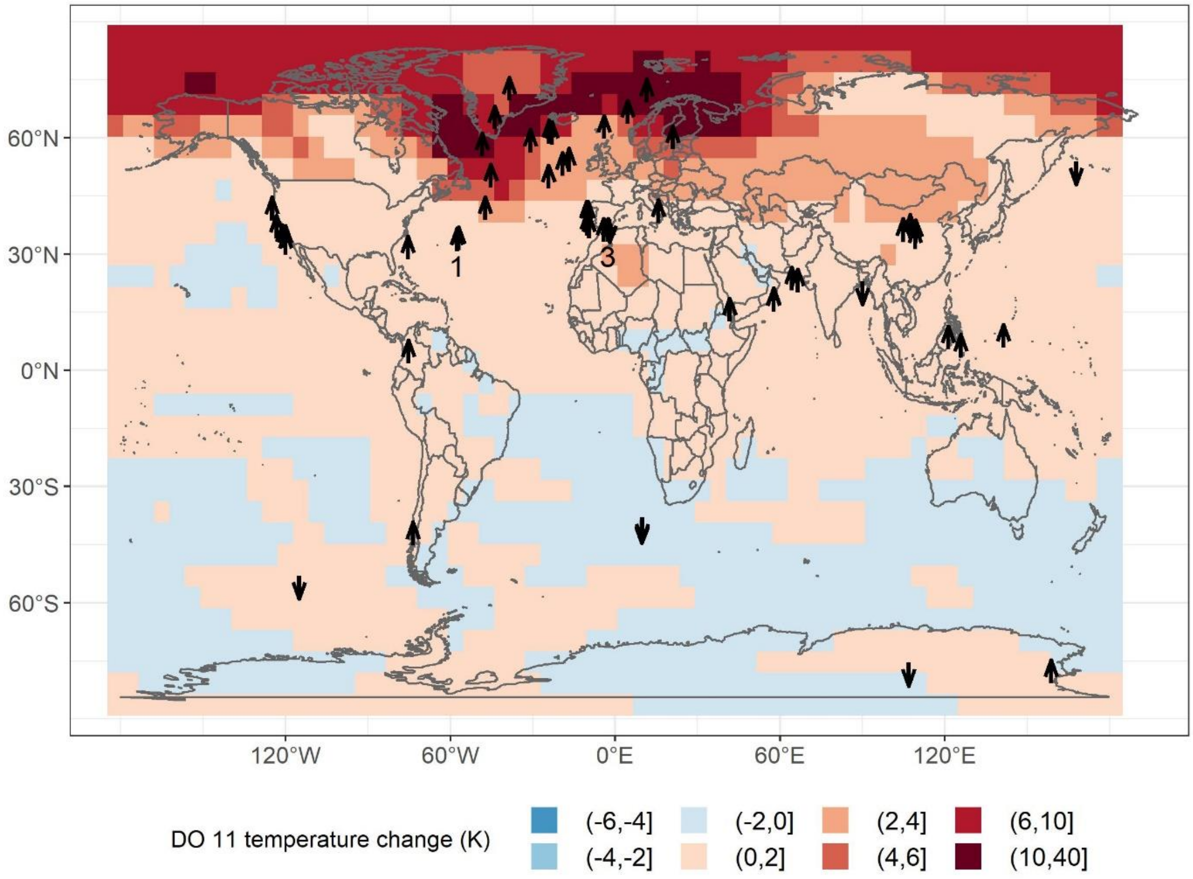

Supplementary Fig. 2.8. Comparison of simulated and observed temperature change during D-O event 12. The coloured map shows the simulated global temperature change pattern. The arrow shows the observed trend (warming or cooling) during all D-O cycles in the record. The text (if any) shows the quantitative temperature change for each D-O cycle in the record.

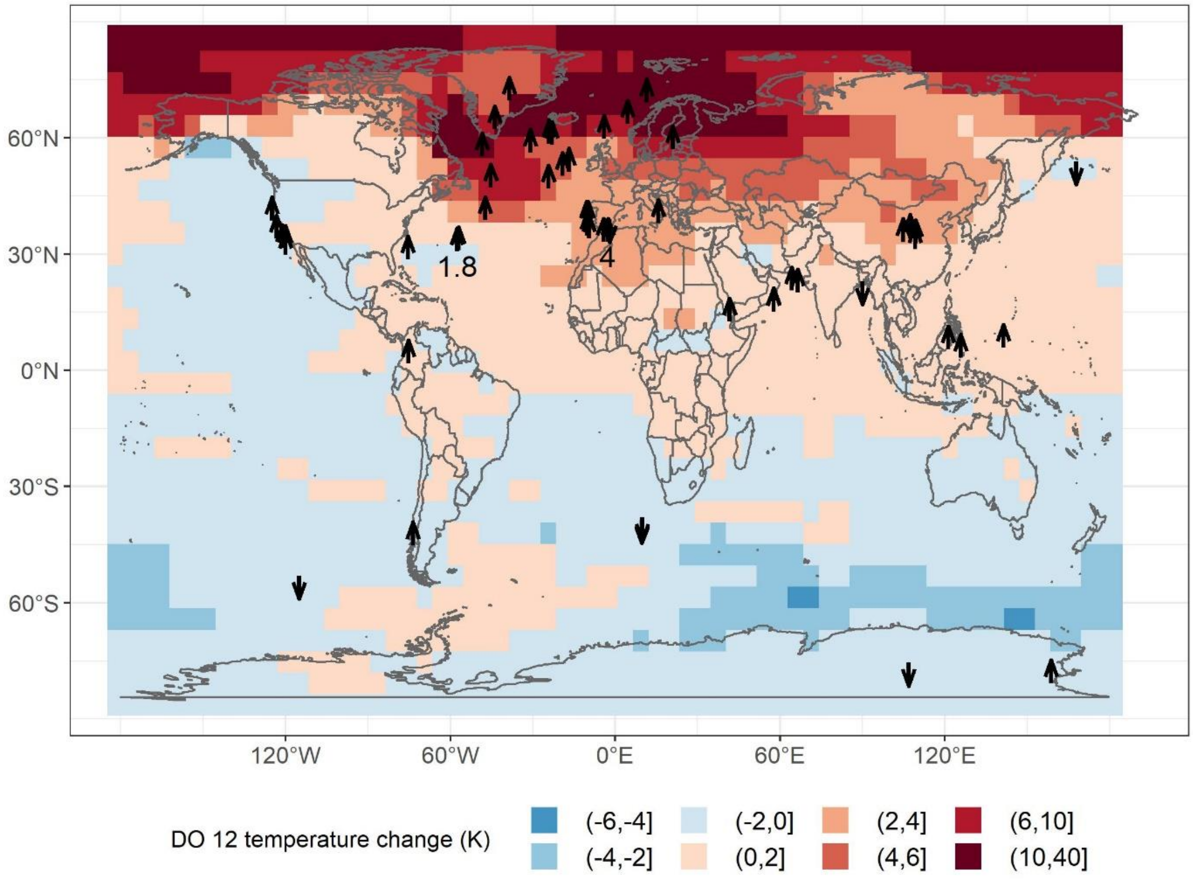

Supplementary Fig. 2.9. Global mean land (black) and ocean (red) temperature anomaly to 30 ka. The data were obtained from LOVECLIM simulations and binned in 25 years. Both the global mean land and ocean temperature were area-weighted, using the cosine of latitude as a weight for each grid. The age is at absolute time scale. The vertical lines show the official start dates of the numbered D-O warming events on AICC2012 timescale (BP 1950).

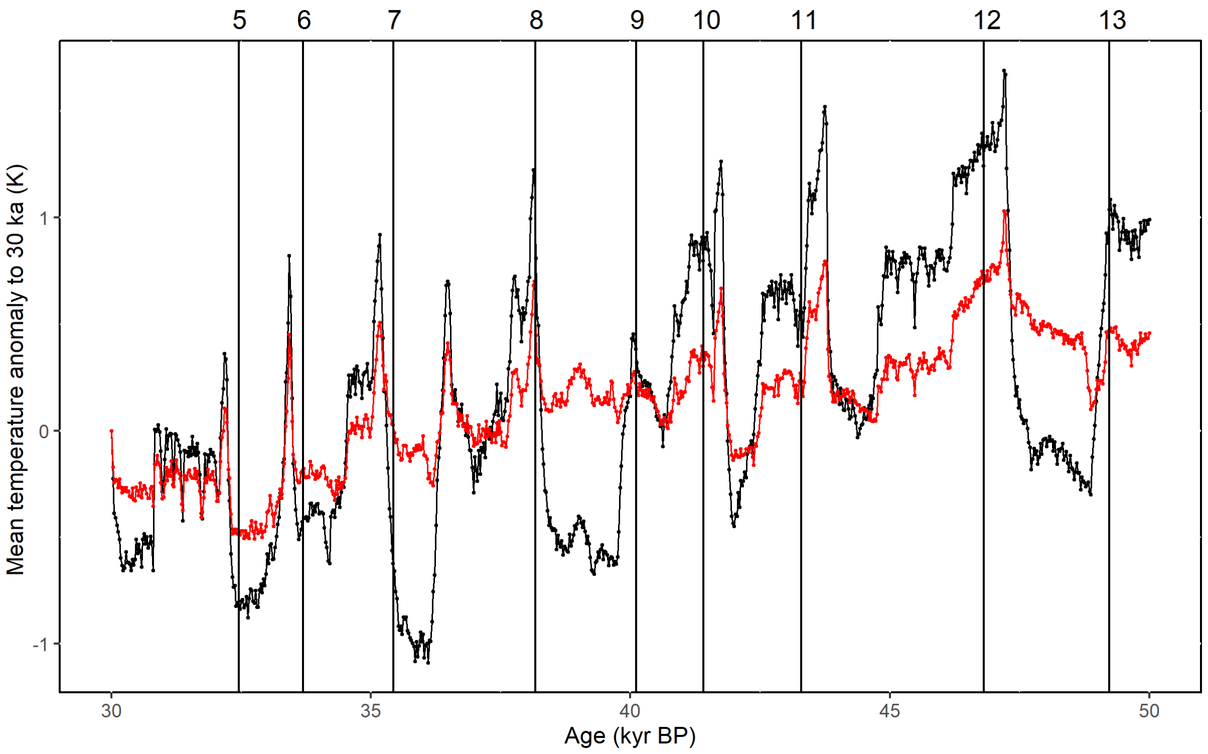

Supplementary Tables

Supplementary Table 1. Sources of ice core data on atmospheric CO_2, CH_4, N_2O, Greenland temperature, δD excess and the start dates of Dansgaard-Oeschger warming events. These data are converted to a common timescale: AICC2012, years before 1950 AD.

Data	Ice core	Source	Reference	Original age model
CO_2	WD ice core	https://www.ncei.noaa.gov/pub/data/paleo/icecore/antarctica/wais2021co2.txt	Bauska *et al.* (2021)	WD2014 (= 1.0063 × GICC05), years before 1950 AD
CH_4	North Greenland Eemian ice core	https://www.ncei.noaa.gov/pub/data/paleo/icecore/greenland/summit/neem/neem2013ch4wscrds-mean.txt	Chappellaz *et al.* (2013)	GICC05, years before 2000 AD
N_2O	NGRIP ice core	https://www.ncei.noaa.gov/pub/data/paleo/icecore/greenland/summit/ngrip/gases/ngrip2013n2o.txt	Schilt *et al.* (2010)	AICC2012, years before 1950 AD
Greenland temperature	NGRIP ice core	https://www.ncei.noaa.gov/pub/data/paleo/icecore/greenland/summit/ngrip/ngrip2014temp.txt	Kindler *et al.* (2014)	GICC05, years before 2000 AD
δD excess	EDC ice core	https://www.ncei.noaa.gov/pub/data/paleo/icecore/antarctica/stenni2010edc-deutex.txt	Stenni *et al.* (2010)	EDC3, years before 1950 AD
Timing of the Dansgaard-Oeschger events	/	/	Wolff *et al.* (2010)	GICC05, years before 2000 AD

Supplementary Table 2. The amplitude and rate of global mean temperature change during each of the numbered D-O cycles.

DO	Global mean temperature change (K)	Time taken (year)	Global mean temperature change rate (K/year)
5	0.773	375	0.0021
6	0.874	175	0.0050
7	0.988	425	0.0023
8	0.831	200	0.0042
9	0.292	500	0.0006
10	1.057	225	0.0047
11	0.850	150	0.0057
12	0.779	225	0.0035

Supplementary Table 3. Feedback strengths and gains with their standard errors.

source	paper	variable	c	σ(c)	g	σ(g)
D-O events	This paper	CO_2	0.155	0.018	0.133	0.048
		CH_4	0.114	0.007	0.099	0.034
		N_2O	0.106	0.013	0.091	0.033
Models	Friedlingstein *et al*. (2006)	CO_2	0.621	/	0.310	/
		CO_2	0.305	/	0.150	/
		CO_2	0.110	/	0.060	/
		CO_2	0.139	/	0.040	/
		CO_2	0.322	/	0.200	/
		CO_2	0.194	/	0.100	/
		CO_2	0.470	/	0.210	/
		CO_2	0.330	/	0.140	/
		CO_2	0.420	/	0.200	/
		CO_2	0.249	/	0.100	/
		CO_2	0.373	/	0.130	/
	Arora *et al*. (2013)	CO_2	0.276	/	0.110	/
		CO_2	0.206	/	0.080	/
		CO_2	0.269	/	0.090	/
		CO_2	0.130	/	0.050	/
		CO_2	0.293	/	0.110	/
		CO_2	0.279	/	0.120	/
		CO_2	0.089	/	0.030	/
		CO_2	0.102	/	0.030	/
		CO_2	0.362	/	0.180	/
	Arora *et al*. (2020)	CO_2	0.175	/	0.072	/
		CO_2	0.499	/	0.183	/
		CO_2	−0.004	/	−0.002	/
		CO_2	0.115	/	0.055	/
		CO_2	0.292	/	0.140	/
		CO_2	0.082	/	0.042	/
		CO_2	0.306	/	0.101	/
		CO_2	0.093	/	0.036	/
		CO_2	0.346	/	0.112	/
		CO_2	0.143	/	0.044	/
		CO_2	0.192	/	0.105	/
	Xu-Ri *et al*. (2012)	N_2O	0.110	0.000	0.095	0.032
	Stocker *et al*. (2013)	CO_2	0.079	0.000	0.068	0.023
		CH_4	0.011	0.000	0.009	0.003
		N_2O	0.023	0.000	0.020	0.007
	IPCC AR6	CH_4	0.030	0.010	0.026	0.012
		N_2O	0.012	0.010	0.010	0.009
Modern	Gedney *et al*. (2019)	CH_4	0.060	0.026	0.052	0.028
Little Ice Age	Scheffer *et al*. (2006) using Moberg *et al*.	CO_2	0.164	0.042	0.133	0.034

	Scheffer *et al*. (2006) using Mann & Jones	CO_2	0.542	0.139	0.439	0.112
	Scheffer *et al*. (2006) recalculation	CO_2	0.335	0.097	0.288	0.112
	Cox & Jones (2008) using Moberg *et al*.	CO_2	0.529	0.135	0.456	0.194
	Khalil & Rasmussen (1989)	CH_4	0.041	0.015	0.035	0.018
		N_2O	0.033	0.012	0.029	0.014
	Khalil & Rasmussen (1989) recalculation	CH_4	0.095	0.038	0.082	0.043
		N_2O	0.077	0.031	0.067	0.035

Supplementary Table 4. Count of the grid cells with the same warming/cooling trend between LOVECLIM simulated global mean temperature change and Voelker (2002) observed global mean temperature change for each D-O event.

D-O	Agreement number	Total number
5	50	58
6	49	58
7	48	58
8	49	58
9	44	58
10	52	58
11	53	58
12	48	58

Supplementary Notes

Carbon-concentration and carbon-climate feedbacks

Here we demonstrate that the CO_2 feedback calculated in this paper, directly based on the observed change in CO_2 concentration during D-O warming events, can be compared to the carbon-climate feedback as it has been calculated in the C^4MIP, CMIP5 and CMIP ensembles: that is, based on the difference between a fully coupled model run forced by rising CO_2, and a model run in which rising CO_2 influences land and ocean carbon uptake but not climate.

1. Feedbacks induced by CO_2 emissions

We first revisit the formalism introduced by Friedlingstein *et al.* (2006)[8] for analysis of the C^4MIP model runs.

No feedback. If all CO_2 emissions (E) remained in the atmosphere, the change in atmospheric CO_2 concentration ($\Delta C_{emitted}$) could be represented by

$$E = m\Delta C_{emitted} \qquad (S1)$$

$$\Delta C_{emitted} = \frac{E}{m} \qquad (S2)$$

where m is the conversion factor from amount to concentration.

Carbon-concentration feedback. If some of the emissions are absorbed by the land and ocean (in other words there is a carbon-concentration feedback), then the change in atmospheric CO_2 concentration (ΔC_C) can be represented by

$$E = \Delta S_{C,land} + \Delta S_{C,ocean} + m\Delta C_C \qquad (S3)$$

where $\Delta S_{C,land}$ ($\Delta S_{C,ocean}$) are the land (ocean) carbon storage changes, both functions of ΔC_C:

$$\Delta S_{C,land} = \beta_L \Delta C_C \qquad (S4)$$

$$\Delta S_{C,ocean} = \beta_O \Delta C_C \qquad (S5)$$

and β_L (β_O) are the land (ocean) carbon sensitivities to atmospheric CO_2 concentration. Therefore,

$$E = (\beta_L + \beta_O + m)\Delta C_C \qquad (S6)$$

Rearranging this equation gives

$$\Delta C_C = \frac{E}{\beta_L + \beta_O + m} \qquad (S7)$$

The gain of the carbon-concentration feedback (g_C) is defined as

$$g_C = \frac{\Delta C_C - \Delta C_{emitted}}{\Delta C_C} \quad (S8)$$

Combining equations (S2), (S7) and (S8) gives

$$g_C = \frac{-(\beta_L + \beta_O)}{m} \quad (S9)$$

β_L and β_O are usually positive, so $g_C < 0$.

Carbon-concentration and carbon-climate feedback. If some of the emissions are absorbed by the land and ocean and the temperature also increases (in other words both carbon-concentration and carbon-climate feedbacks operate), then the change in atmospheric CO_2 concentration (ΔC_{CT}) can be represented by

$$E = \Delta S_{CT,land} + \Delta S_{CT,ocean} + m\Delta C_{CT} \quad (S10)$$

where

$$\Delta S_{CT,land} = \beta_L \Delta C_{CT} + \gamma_L \Delta T_{CT} \quad (S11)$$

$$\Delta S_{CT,ocean} = \beta_O \Delta C_{CT} + \gamma_O \Delta T_{CT} \quad (S12)$$

and γ_L (γ_O) are the land (ocean) carbon sensitivities to temperature. Therefore,

$$E = (\beta_L + \beta_O)\Delta C_{CT} + (\gamma_L + \gamma_O)\Delta T_{CT} + m\Delta C_{CT} \quad (S13)$$

Rearranging this equation gives

$$\Delta C_{CT} = \frac{E - (\gamma_L + \gamma_O)\Delta T_{CT}}{\beta_L + \beta_O + m} \quad (S14)$$

Representing the effect of changing CO_2 on global mean temperature[8] as

$$\Delta T_{CT} = \alpha \Delta C_{CT} \quad (S15)$$

gives

$$\Delta C_{CT} = \frac{E}{\beta_L + \beta_O + m + (\gamma_L + \gamma_O)\alpha} \quad (S16)$$

Friedlingstein *et al.* (2006)[8] used the difference between the fully coupled model and the model with only carbon-concentration feedback to estimate the gain of the carbon-climate feedback (g_T):

$$g_T = \frac{\Delta C_{CT} - \Delta C_C}{\Delta C_{CT}} \quad (S17)$$

Combining equations (S7), (S16) and (S17) gives

$$g_T = \frac{-(\gamma_L + \gamma_O)\alpha}{\beta_L + \beta_O + m} \qquad (S18)$$

β_L and β_O are usually positive and γ_L and γ_O are usually negative, so $g_T > 0$.

Climate-induced carbon-concentration feedback. When temperature increases (assuming $\gamma_L + \gamma_O < 0$), atmospheric CO_2 also increases, and this increase is moderated (assuming $\beta_L + \beta_O > 0$) by the carbon-concentration feedback. We use the term ΔC_{CT-Cc} to represent the change in CO_2 concentration due to both carbon-concentration and carbon-climate feedbacks that would occur in the absence of this moderating effect:

$$g_C = \frac{\Delta C_{CT} - \Delta C_{CT-Cc}}{\Delta C_{CT}} \qquad (S19)$$

Rearranging this equation gives

$$\Delta C_{CT-Cc} = (1 - g_C)\Delta C_{CT} \qquad (S20)$$

Combining equations (S9) and (S16) gives

$$\Delta C_{CT-Cc} = \left(1 + \frac{\beta_L + \beta_O}{m}\right)\frac{E}{\beta_L + \beta_O + m + (\gamma_L + \gamma_O)\alpha} = \frac{\frac{E}{m}}{1 + \frac{(\gamma_L + \gamma_O)\alpha}{\beta_L + \beta_O + m}} \qquad (S21)$$

Combining equations (S2), (S18) and (S21) gives

$$\Delta C_{CT-Cc} = \frac{\Delta C_{emitted}}{1 - g_T} \qquad (S22)$$

Rearranging this equation gives

$$g_T = \frac{\Delta C_{CT-Cc} - \Delta C_{emitted}}{\Delta C_{CT-Cc}} \qquad (S23)$$

Applying equation (S15) to represent the CO_2 effect on climate:

$$\Delta T_{CT-Cc} = \alpha \Delta C_{CT-Cc} \qquad (S24)$$

$$\Delta T_{emitted} = \alpha \Delta C_{emitted} \qquad (S25)$$

Therefore,

$$g_T = \frac{\Delta T_{CT-Cc} - \Delta T_{emitted}}{\Delta T_{CT-Cc}} \qquad (S26)$$

Equation (S26) implies that the temperature increase ($\Delta T_{emitted}$) is amplified through carbon-climate feedback, resulting in a new temperature increase ΔT_{CT-Cc}.

The "pure" carbon-climate feedback. If elevated atmospheric CO_2 causes a temperature increase but there is no land and ocean carbon uptake, the change in atmospheric CO_2 concentration (ΔC_T) can be represented by

$$E = \Delta S_{T,land} + \Delta S_{T,ocean} + m\Delta C_T \quad (S27)$$

where

$$\Delta S_{T,land} = \gamma_L \Delta T_T \quad (S28)$$

$$\Delta S_{T,ocean} = \gamma_O \Delta T_T \quad (S29)$$

Therefore,

$$E = (\gamma_L + \gamma_O)\Delta T_T + m\Delta C_T \quad (S30)$$

Rearranging this equation gives

$$\Delta C_T = \frac{E - (\gamma_L + \gamma_O)\Delta T_T}{m} \quad (S31)$$

Assuming

$$\Delta T_T = \alpha \Delta C_T \quad (S32)$$

gives

$$\Delta C_T = \frac{E}{m + (\gamma_L + \gamma_O)\alpha} \quad (S33)$$

The gain of the "pure climate feedback" ($g_{T'}$) can now be defined as

$$g_{T'} = \frac{\Delta C_T - \Delta C_{emitted}}{\Delta C_T} \quad (S34)$$

Combining equations (S2), (S33) and (S34) gives

$$g_{T'} = \frac{-(\gamma_L + \gamma_O)\alpha}{m} \quad (S35)$$

Unlike equation (S18), this gain does not contain concentration feedback terms (β_L, β_O).

The combined gain of the carbon-climate feedback and carbon-concentration feedbacks is

$$g_{CT} = \frac{\Delta C_{CT} - \Delta C_{emitted}}{\Delta C_{CT}} \quad (S36)$$

Rearranging this equation gives

$$\Delta C_{CT} = \frac{\Delta C_{emitted}}{1 - g_{CT}} \qquad (S37)$$

Rearranging equations (S8) and (S17) gives

$$\Delta C_{C} = \frac{\Delta C_{emitted}}{1 - g_{C}} \qquad (S38)$$

$$\Delta C_{CT} = \frac{\Delta C_{C}}{1 - g_{T}} \qquad (S39)$$

Combining equations (S38) and (S30) gives

$$\Delta C_{CT} = \frac{\Delta C_{emitted}}{(1 - g_{C})(1 - g_{T})} \qquad (S40)$$

From equations (S37) and (S40)

$$1 - g_{CT} = (1 - g_{C})(1 - g_{T}) \qquad (S41)$$

$$g_{CT} = g_{C} + g_{T} - g_{C}g_{T} \qquad (S42)$$

Therefore, the carbon-climate feedback and carbon-concentration feedback as defined in Friedlingstein *et al.* (2006)[8] do not add up to the total carbon feedback.

According to equations (S9) and (S18), however,

$$g_{T} - g_{C}g_{T} = \frac{-(\gamma_{L} + \gamma_{O})\alpha}{m} \qquad (S43)$$

This is the same as equation (S35), so

$$g_{T'} = g_{T} - g_{C}g_{T} \qquad (S44)$$

Therefore,

$$g_{CT} = g_{C} + g_{T'} \qquad (S45)$$

In other words, the carbon-concentration feedback and the “pure” carbon-climate feedback do add up to the total carbon feedback.

Equation (S44) can also be written as

$$g_{T} = \frac{g_{T'}}{1 - g_{C}} \qquad (S46)$$

which clarifies that the carbon-climate feedback as defined in Friedlingstein *et al.* (2006)[8] contains both the “pure” climate feedback and the climate-induced concentration feedback.

2. Feedbacks induced by D-O warming events

D-O warming events drive changes in atmospheric CO_2. The amount of CO_2 released to the atmosphere due to this warming is

$$\Delta C_{actual-Cc} = \frac{-(\gamma_L + \gamma_O)\Delta T}{m} \qquad (S47)$$

However, not all of this CO_2 will remain in the atmosphere, as some will be taken up by climate-induced concentration feedback. According to equation (S20), the resulting net release of CO_2 will be

$$\Delta C_{actual} = \frac{\frac{-(\gamma_L + \gamma_O)\Delta T}{m}}{(1 - g_C)} = \frac{-(\gamma_L + \gamma_O)\Delta T}{m + \beta_L + \beta_O} \qquad (S48)$$

Here, we calculate gain as the product of the climate sensitivity and the response of CO_2 to temperature:

$$g = \alpha \frac{\Delta C_{actual}}{\Delta T} \qquad (S49)$$

Combining equations (S48) and (S49) gives

$$g = \frac{-(\gamma_L + \gamma_O)\alpha}{m + \beta_L + \beta_O} \qquad (S50)$$

This equation is the same as equation (S18), so

$$g = g_T \qquad (S51)$$

We conclude that the gain as calculated in this paper is identical with the gain of the carbon-climate feedback as defined by Friedlingstein *et al.* (2006)[8].

Potential extension of the period using Greenland ice core data

There is a strong relationship between global mean temperature change and north-eastern Greenland temperature change in LOVECLIM outputs (correlation coefficient= 0.86, $p < 0.001$), as shown in Supplementary Fig. E1.

Supplementary Fig. E1. The relationship between global mean temperature anomaly to 30 ka and north-eastern Greenland temperature to 30 ka in LOVECLIM outputs.

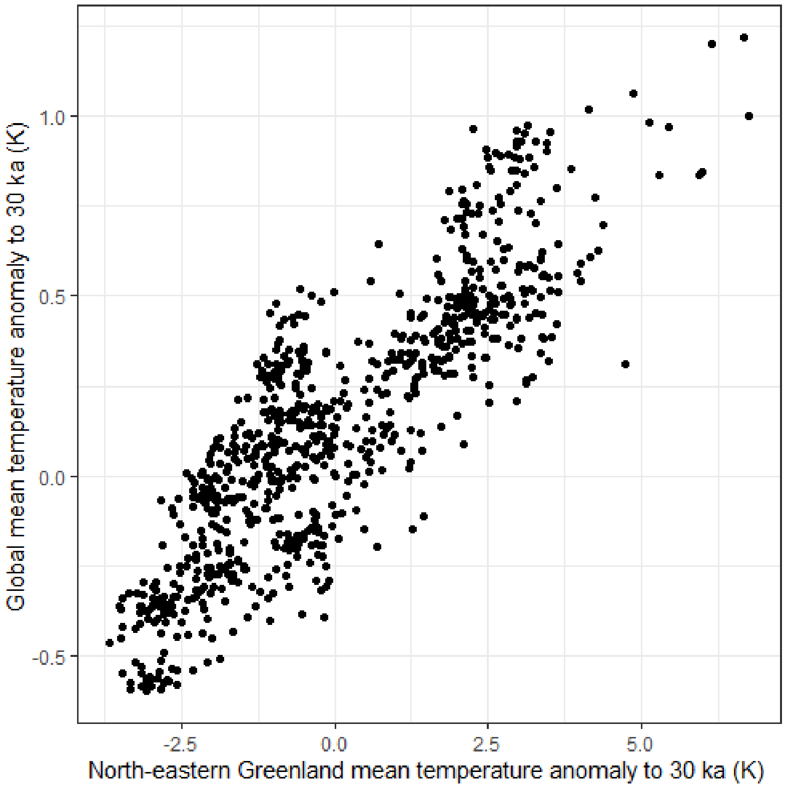

There is also a highly significant relationship (correlation coefficient= 0.91) between global mean temperature and north-eastern Greenland temperature at present-day (Chylek, P. & Lohmann, U. Ratio of the Greenland to global temperature change: Comparison of observations and climate modeling results. *Geophys. Res. Lett.* **32**, (2005).)

If we assume this relationship to be robust, we can use the identified NGRIP temperature change to extend the period from DO 5 ~ 12 (50 ~ 30 ka) to DO 2 ~ 20 (80 ~ 20 ka).

Supplementary Fig. E2. The relationship between simulated global mean temperature change and observed north-eastern Greenland (NGRIP) temperature change during D-O 5 ~12. The vertical lines show 95% confidence intervals of simulated global mean temperature changes.

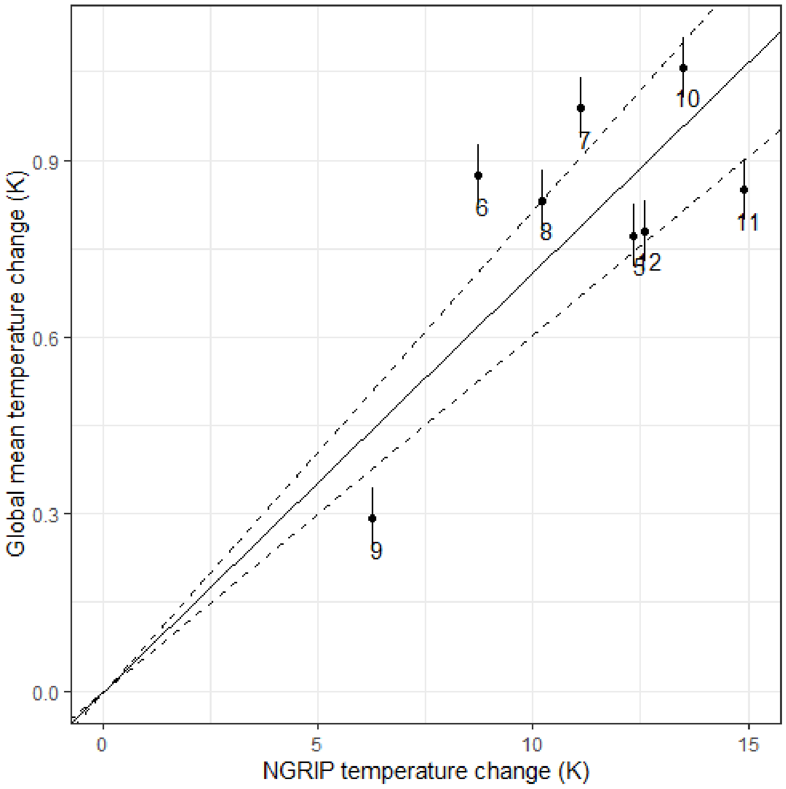

The ratio (b) of simulated global mean temperature change to NGRIP temperature change, as shown in Supplementary Fig. E2, and the standard error of the ratio (σ_b), were obtained by doing a linear regression without intercept, with $1/\sigma_y^2$ as weights, since there was no error provided for NGRIP temperature in the data source.

The global mean temperature change during D-O 2 ~ 20 can then be obtained by

$$\Delta T_{mean\ global} = b\ \Delta T_{\mathrm{NGRIP}}$$

with the standard error

$$\sigma_{\Delta T_{mean\ global}} = \sigma_b\ \Delta T_{\mathrm{NGRIP}}$$

We use the same procedure as in the main text, and get the estimation of feedback strengths and the gain (Supplementary Fig. E3, Supplementary Table E1). We also compare the gain with previous attempts in Supplementary Fig. E4.

Supplementary Fig. E3. Maximum likelihood estimation of feedback strengths, using D-O events 2~20. The figure shows the relationship between the increase in global mean temperature and radiative forcing induced by changes in (a) CO_2, (b) CH_4, (c) N_2O concentrations and (d) combined radiative forcing of CO_2, CH_4 and N_2O. Each D-O event is numbered; the horizontal and vertical lines show the 95% confidence intervals. The measurements of CH_4 concentration are very accurate so the vertical lines are too small to be observable on these plots. The solid line shows the maximum likelihood estimation of the ratio of radiative forcing to global mean temperature increase, the dashed lines show the 95% confidence intervals of the ratio.

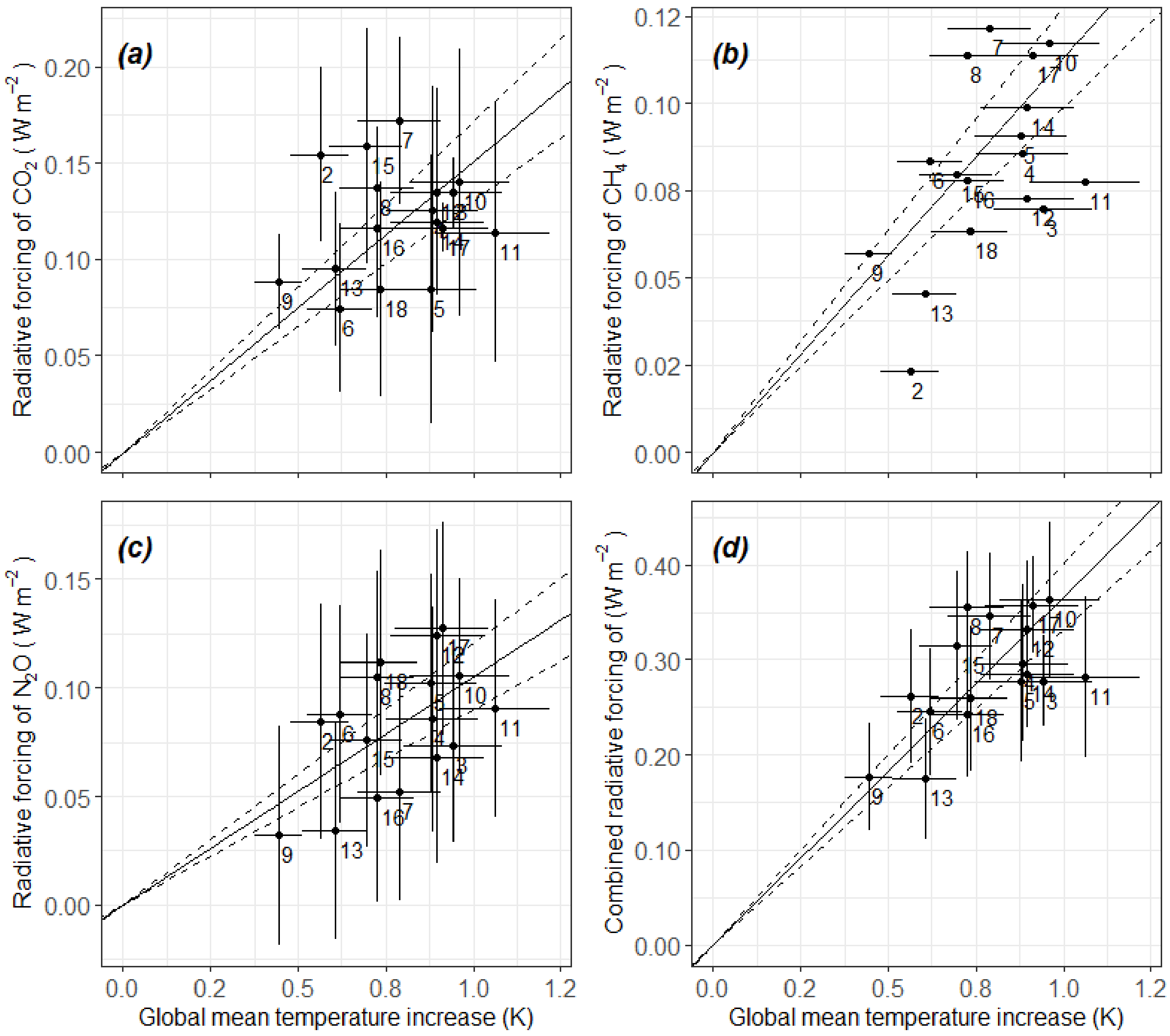

Supplementary Table E1. Feedbacks estimated from D-O events 2~20. *c* is the feedback strength, *g* is the gain, while σ_c is the standard error of the feedback strength and σ_g is the standard error of the gain.

	c (W m^{-2} K^{-1})	σ_c (W m^{-2} K^{-1})	g	σ_g
CO_2	0.152	0.011	0.131	0.046
CH_4	0.113	0.007	0.097	0.034
N_2O	0.105	0.008	0.091	0.032
Combined	0.366	0.018	0.316	0.109
